# Evaluation of near point‐of‐care viral load implementation in public health facilities across seven countries in sub‐Saharan Africa

**DOI:** 10.1002/jia2.25663

**Published:** 2021-01-16

**Authors:** Caroline E Boeke, Jessica Joseph, Charles Atem, Clement Banda, Khady Diatou Coulibaly, Naoko Doi, Andrews Gunda, James Kandulu, Brianán Kiernan, Leonard Kingwara, Werner Maokola, Tatenda Maparo, Rose Nadege Mbaye, Esther Mtumbuka, Joseph Mziray, Catherine Ngugi, Jeanine Nkakulu, Divine Nzuobontane, Marie Claire Okomo Assoumo, Trevor Peter, Maria R Rioja, Jilian A Sacks, Raiva Simbi, Lara Vojnov, Shaukat A Khan

**Affiliations:** ^1^ Clinton Health Access Initiative Boston MA USA; ^2^ Clinton Health Access Initiative Yaounde Cameroon; ^3^ Clinton Health Access Initiative Lilongwe Malawi; ^4^ Division de la Lutte Contre le SIDA et les IST Ministère de la Santé et de l’Action Sociale Dakar Senegal; ^5^ Ministry of Health and Population Lilongwe Malawi; ^6^ Clinton Health Access Initiative Dakar Senegal; ^7^ NASCOP SI unit National HIV Reference Laboratory Nairobi Kenya; ^8^ MOHCDGEC‐NACP Dar Es Salaam Tanzania; ^9^ Clinton Health Access Initiative Harare Zimbabwe; ^10^ Clinton Health Access Initiative Dar Es Salaam Tanzania; ^11^ National AIDS and STI Control Programme (NASCOP) Nairobi Kenya; ^12^ Clinton Health Access Initiative Kinshasa Democratic Republic of Congo; ^13^ National Public Health Laboratory Yaounde Cameroon; ^14^ Ministry of Health and Child Care Harare Zimbabwe

**Keywords:** point of care, viral load monitoring, Africa, viral suppression

## Abstract

**Introduction:**

In many low‐ and middle‐income countries, HIV viral load (VL) testing occurs at centralized laboratories and time‐to‐result‐delivery is lengthy, preventing timely monitoring of HIV treatment adherence. Near point‐of‐care (POC) devices, which are placed within health facility laboratories rather than clinics themselves (i.e. “true” POC), can offer VL in conjunction with centralized laboratories to expedite clinical decision making and improve outcomes, especially for patients at high risk of treatment failure. We assessed impacts of near‐POC VL testing on result receipt and clinical action in public sector programmes in Cameroon, Democratic Republic of Congo, Kenya, Malawi, Senegal, Tanzania and Zimbabwe.

**Methods:**

Routine health data were collected retrospectively after introducing near‐POC VL testing at 57 public sector health facilities (2017 to 2019, country‐dependent). Where possible, key indicators were compared to data from patients receiving centralized laboratory testing using hazard ratios and the Somers’ D test.

**Results:**

Data were collected from 6795 tests conducted on near‐POC and 17614 tests on centralized laboratory‐based platforms. Thirty‐one percent (2062/6694) of near‐POC tests were conducted for high‐risk populations: pregnant and breastfeeding women, children and those with suspected failure. Compared to conventional testing, near‐POC improved the median time from sample collection to return of results to patient [six vs. sixty‐eight days, effect size: −32.2%; 95% CI: −41.0% to −23.4%] and to clinical action for individuals with an elevated HIV VL [three vs. fourty‐nine days, effect size: −35.4%; 95% CI: −46.0% to −24.8%]. Near‐POC VL results were two times more likely to be returned to the patient within 90 days compared to centralized tests [50% (1781/3594) vs. 27% (4172/15271); aHR: 2.22, 95% CI: 2.05 to 2.39]. Thirty‐seven percent (340/925) of patients with an elevated near‐POC HIV VL result had documented clinical follow‐up actions within 30 days compared to 7% (167/2276) for centralized testing.

**Conclusions:**

Near‐POC VL testing enabled rapid test result delivery for high‐risk populations and led to significant improvements in the timeliness of patient result receipt compared to centralized testing. While there was some improvement in time‐to‐clinical action with near‐POC VL testing, major gaps remained. Strengthening of systems supporting the utilization of results for patient management are needed to truly capitalize on the benefits of decentralized testing.

## 1 INTRODUCTION

HIV viral load (VL) monitoring is recommended by the World Health Organization (WHO) to confirm viral suppression and to take action if a patient has an elevated VL [1]. Clinicians and patients must have access to timely VL results to take clinical actions should VL be elevated. Controlling virus levels to undetectable improves patient health and reduces the risk of onward transmission [2, 3, 4]. HIV VL coverage has scaled up substantially over time and in 2018 it was estimated that among those in need of a VL test in low‐ and middle‐income countries (LMIC), 66% received one [5]. However, despite large financial investments in HIV VL testing, test results are often not returned to clinicians and patients and clinical utilization of results remains low [6, 7, 8].

Near point‐of‐care (near‐POC) HIV VL technologies are simpler to operate and do not require the infrastructure of a full laboratory. Near‐POC devices are placed within health facility laboratories rather than directly within the clinic where the patient is being seen (“true” POC). This technology theoretically may enable decentralization of testing to lower levels of health facilities, thus eliminating the need for sample transportation for many patients, and can deliver test results within hours of sample collection. As a result, near‐POC VL testing has the potential to address some of the current gaps in VL monitoring including improving and expediting result return to both clinicians and patients and clinical utilization [9]. Therefore, near‐POC VL may facilitate better patient management and improve rates of viral suppression. While near‐POC may allow for shorter time‐to‐results, the daily throughput of near‐POC devices is limited; the four‐module devices found in most LMICs can run approximately 20 tests per 8‐hour workday. Testing strategies may consider near‐POC devices in conjunction with centralized laboratory testing to address demand and prioritize samples for high‐risk patients.

To date, there is limited research on how best to implement near‐POC testing as part of a national HIV VL network or testing strategy in LMICs, the impact of near‐POC VL on patient care, or the management of specific patient groups that could benefit most from near‐POC VL testing [10, 11, 12, 13]. To better understand the operational performance of near‐POC VL testing in resource‐limited settings, we assessed key aspects of routine near‐POC testing programmes at 57 facilities across seven sub‐Saharan African countries, including time from sample collection to patient receipt of results and utilization of test results for clinical management.

## 2 METHODS

### 2.1 Study design

Delivery of testing services and clinical outcomes was assessed after the introduction of near‐POC VL at 57 public sector health facilities as part of service delivery programmes in seven countries: Cameroon, Democratic Republic of Congo (DRC), Kenya, Malawi, Senegal, Tanzania and Zimbabwe. In Senegal and Zimbabwe, some data were collected as part of ongoing studies with information that could be used for this analysis. Data were collected from May 2017‐October 2019 (country‐dependent). Descriptions of the assessment structure by country and study design can be found in Table 1.

**Table 1:** Near‐POC HIV VL implementation by country and study design

	Cameroon	DRC	Kenya	Malawi	Senegal – 1	Senegal – 2	Tanzania	Zimbabwe – 1	Zimbabwe – 2
Number of facilities	3	4	4	10	4	4	10	8	10
Facility selection	All facilities with near‐POC	All facilities with near‐POC	First 4 sites that rolled out near‐POC	All facilities with near‐POC	Facilities with GeneXpert, high HIV patient load and low TB test volumes	All facilities with mPIMA	Random selection of facilities with near‐POC	All facilities with near‐POC	All facilities with near‐POC not included in Zimbabwe‐1
Study design	Near‐POC only	Near‐POC only	Pre/post	Cross‐sectional^a^	Near‐POC only	Pre/post	Cross‐sectional^a^	Pre/post	Cross‐sectional^a^
POC Device	GeneXpert	GeneXpert	GeneXpert	GeneXpert	GeneXpert	mPIMA	GeneXpert	GeneXpert	GeneXpert
Time frame									
Centralized testing period	–	–	Oct 2017 to Jun 2018	Jun 2017 to Sep 2017	–	Jul 2018 to Oct 2018	Jan to Jun 2018	May 2017 to Sep 2017	Oct 2018 to Mar 2019
POC testing period	Nov 2018 to Apr 2019	Jun to Dec 2018	Oct 2018 to Jun 2019	Jun 2017 to Sep 2017	May to Jul 2019	Jul 2019 to Oct 2019	Jan to Jun 2018	Oct 2017 to Feb 2018	Oct 2018 to Mar 2019
Length of implementation period	6 months	7 months	9 months	4 months	3 months	3 months	6 months	5 months	6 months
Length of follow‐up	90 days	90 days	90 days	90 days	30 days	30 days	90 days	90 days	90 days

DRC, Democratic Republic of Congo; POC, point‐of‐care; VL: viral load.

^a^ Data from VL samples assessed using near‐POC were compared to samples from the same facilities that were assessed using centralized laboratory testing.

National HIV programmes had completed their own processes to select health facilities for near‐POC VL implementation based on criteria such as current device availability, volume of patients on antiretroviral therapy (ART) per facility, history of stockouts and current device utilization rate. For this analysis, 3 to 10 facilities with near‐POC devices were randomly selected within each country. The assessment period ranged from three to nine months per country, depending on logistical factors. Data were retrospectively collected for all patients on ART receiving near‐POC VL testing during the assessment period. Where possible, key indicators were compared to data from all patients on ART receiving VL testing through the centralized laboratory in the three‐ to nine‐month period before near‐POC VL testing was implemented (Kenya, Senegal and 8 facilities in Zimbabwe) or to data from patients receiving centralized laboratory testing at the same facilities during the same period (Malawi, Tanzania and 10 different facilities in Zimbabwe). In Cameroon and DRC, no comparison data were available on centralized testing because there was previously no centralized HIV VL programme.

### 2.2 HIV VL testing guidelines and implementation of near‐POC VL testing at public sector facilities

The 2016 WHO treatment guidelines recommend that patients receive a first HIV VL test at six months post‐ART initiation, a second at 12 months post‐ART initiation and annual tests thereafter [1]. If a person has an elevated VL above 1000 copies/mL, adherence to ART regimen is determined through three consecutive months of “enhanced adherence counselling” (EAC). Adherence counselling, as defined by each country, is a comprehensive approach and includes actions such as adjustment of dosing or working on tolerance based on clinician judgement. If a second, confirmatory VL measurement remains elevated after EAC, drug‐resistance is assumed to have rendered the current treatment ineffective. The patient should be considered for switching to the nationally recommended second‐line ART regimen. All countries involved in this assessment have VL eligibility testing policies aligned with the WHO recommendations. Malawi was an exception which stated that patients who were stable after 12 months (no elevations greater than 1000 copies/mL) received monitoring VL every two years versus annually.

A single Cepheid GeneXpert (Cepheid, Sunnyvale, CA) or Abbott m‐PIMA (Abbott, Chicago, IL) device was available for near‐POC VL testing at the health facilities included in this assessment. Rollout of near‐POC testing included training existing health facility staff on operational procedures for testing, clinical utilization of rapid test results, results documentation, device maintenance, supply chain and waste management and quality control.

Near‐POC VL samples were only collected from patients receiving care at the selected study facilities. In most countries, all patients receiving VL technically were eligible for near‐POC VL testing. However, prioritization of patients at high risk of having viraemia (children, adolescents, patients suspected of treatment failure, most recent VL ≥ 1000 copies/mL) or at high risk of transmission (pregnant and breastfeeding women) occurred in most contexts. Ultimately, testing decisions were made by individual clinics/healthcare workers. In Kenya, near‐POC VL testing was only made available for paediatric and adolescent populations and pregnant and breastfeeding women.

Near‐POC VL devices were placed in the onsite laboratories in the health facilities and were operated by laboratory staff. Centralized VL tests were sent to a district hospital or a National Referral Lab for testing. Whole blood samples were collected by clinic or laboratory staff using venipuncture and were processed for testing per manufacturer’s instruction.

### 2.3 Data collection and analysis procedures

Trained data collectors retrospectively extracted data from the health facilities included in this assessment. Data included demographic information, reason for VL test (i.e. routine monitoring or suspected failure), results, clinical action taken, if any, among patients with an elevated VL, and timing of each step in the cascade from sample collection to clinical action for each patient tested using national and facility‐specific laboratory and clinic registers and patient charts. Electronic data collection using SurveyCTO (2016 Dobility, Inc., Cambridge, Massachusetts) forms on tablets were used. Data collection for both near‐POC VL and centralized VL testing outcomes allowed for a follow‐up period of 90 days to allow for complete documentation of testing and treatment outcomes in facility registers. In Senegal, only 30 days of follow‐up were possible due to funding/logistical constraints.

All samples collected within the study periods were included. Only tests with valid results (i.e. errors, invalid and missing excluded) were included in the main analyses. The primary outcomes were: clinic receipt of test results during follow‐up (documented in clinic records); patient receipt of test results during follow‐up (documented in clinic records); turnaround times between sample collection and receipt of test results by the clinic and patient; and turnaround time to clinical action for patients with a documented elevated VL result. Turnaround time was defined as the number of days between sample collection and the outcome (receipt of results, clinical action), for those with an outcome during follow‐up. Clinical action was defined as either a documented enhanced adherence counselling after a first elevated HIV VL (≥1000 copies/mL) or a switch to second‐line regimen upon a second elevated VL. Shorter timeframes were also assessed for these indicators (7, 30 and 90 days). Patients with missing dates for steps in the cascade of care were considered as failing to complete that step during analysis. In countries without centralized testing comparison data, only descriptive analyses were presented, including percentages and medians with interquartile ranges (IQR).

Continuous outcomes were compared using the Somers’ D test, accounting for facility‐level clustering [14]. Adjusted hazard ratios (aHR) and 95% confidence intervals (CI) were calculated using maximum likelihood estimation for parametric regression survival‐time models to compare time‐to‐event data. The *streg* command in Stata was utilized, accounting for facility‐level clustering using shared frailties. Models were adjusted for study design, sex, age, test reason, viral load suppression and country. Kaplan–Meier curves were constructed to visually compare groups. Statistical analysis was performed using StataSE 15 (StataCorp, College Station, TX, USA).

### 2.4 Ethical approvals

Approval for this study was obtained from a local Institutional Review Board (IRB) in each country where required (Cameroon: Comite National D’Ethique de la Recherche pour la Santé Humaine 2020/08/1587/L/CNERSH/SP; DRC: Université de Kinshasa Comite D’Ethique ESP/CE/079/2019; Kenya: Kenyatta National Hospital – University of Nairobi (KNH‐UoN); Malawi: National Health Sciences Research Committee #1666/Chesapeake IRB Pro00021907; Tanzania: National Institute for Medical Research NIMR/HQ/R.8a/Vol. IX/3188; Zimbabwe‐2: Medical Research Council of Zimbabwe MRCZ/A/2470; and United States: Advarra Institutional Review Board #Pro00030414). Written informed consent was waived by all IRBs as all activities conducted were considered standard and routine practice and datasets did not include personal identifiers.

## 3 RESULTS

Across all countries, 6795 near‐POC VL tests and 17614 centralized VL tests were conducted, of which 31% versus 6% were conducted for high‐risk populations respectively (Table 2). Of the near‐POC VL tests conducted for high‐risk populations, 33% were for patients suspected of treatment failure, 27% were for pregnant and breastfeeding women, and 18% were for children and adolescents; 22% had missing information on the population. Twenty‐four percent of patients tested on near‐POC had an elevated VL compared to 15% of those receiving centralized VL testing, reflecting the prioritization of near‐POC for high‐risk patients. Overall, only 4% of near‐POC tests had missing results compared to 27% of centralized VL tests; error rates were similar (near‐POC: 4%; centralized: 6%). Patient characteristics are shown by study in Table S1.

**Table 2:** Characteristics of patients receiving HIV VL testing by test implementation method

	Near‐POC		Centralized	
	N	n (%)/median (IQR)	N	n (%)/median (IQR)
Total tests	6795		17614	
Study design	6795		17614	
Cross‐sectional		2662 (39%)		13693 (78%)
Pre/post		1729 (25%)		3921 (22%)
Near‐POC only		2404 (35%)		0 (0%)
Age, years	6225	39 (28 to 48)	11061	42 (33 to 50)
Age	6225		11061	
Children (0 to 14 years)		767 (12%)		696 (6%)
Adolescents (15 to 19 years)		233 (4%)		459 (4%)
Adult (20 to 65 years)		5068 (81%)		9526 (86%)
>65 years		157 (3%)		380 (3%)
Female	6759	4801 (71%)	17407	11326 (65%)
Test results	6795		17614	
Valid		6212 (91%)		15378 (67%)
Error		288 (4%)		431 (6%)
Missing		295 (4%)		1805 (27%)
Test reason	6694		17188	
Routine HIV VL monitoring		4454 (67%)		15795 (92%)
Follow‐up HIV VL		178 (3%)		286 (2%)
High‐risk population		2062 (31%)		1107 (6%)
Children/adolescents		380 (18%)		358 (32%)
Pregnant/breastfeeding women		552 (27%)		296 (27%)
Suspected failure		676 (33%)		186 (17%)
Missing reason		454 (22%)		267 (24%)
Elevated HIV VL (≥1000 copies/mL)	6212	1487 (24%)	15378	2276 (15%)
Elevated HIV VL, routine HIV VL monitoring	4157	747 (18%)	13764	1672 (12%)
Elevated HIV VL, follow‐up HIV VL	170	114 (67%)	257	137 (53%)
Elevated HIV VL, high‐riskpopulation	1791	593 (33%)	988	374 (38%)

IQR, interquartile range; POC, point‐of‐care; VL, viral load.

**Figure 1:**
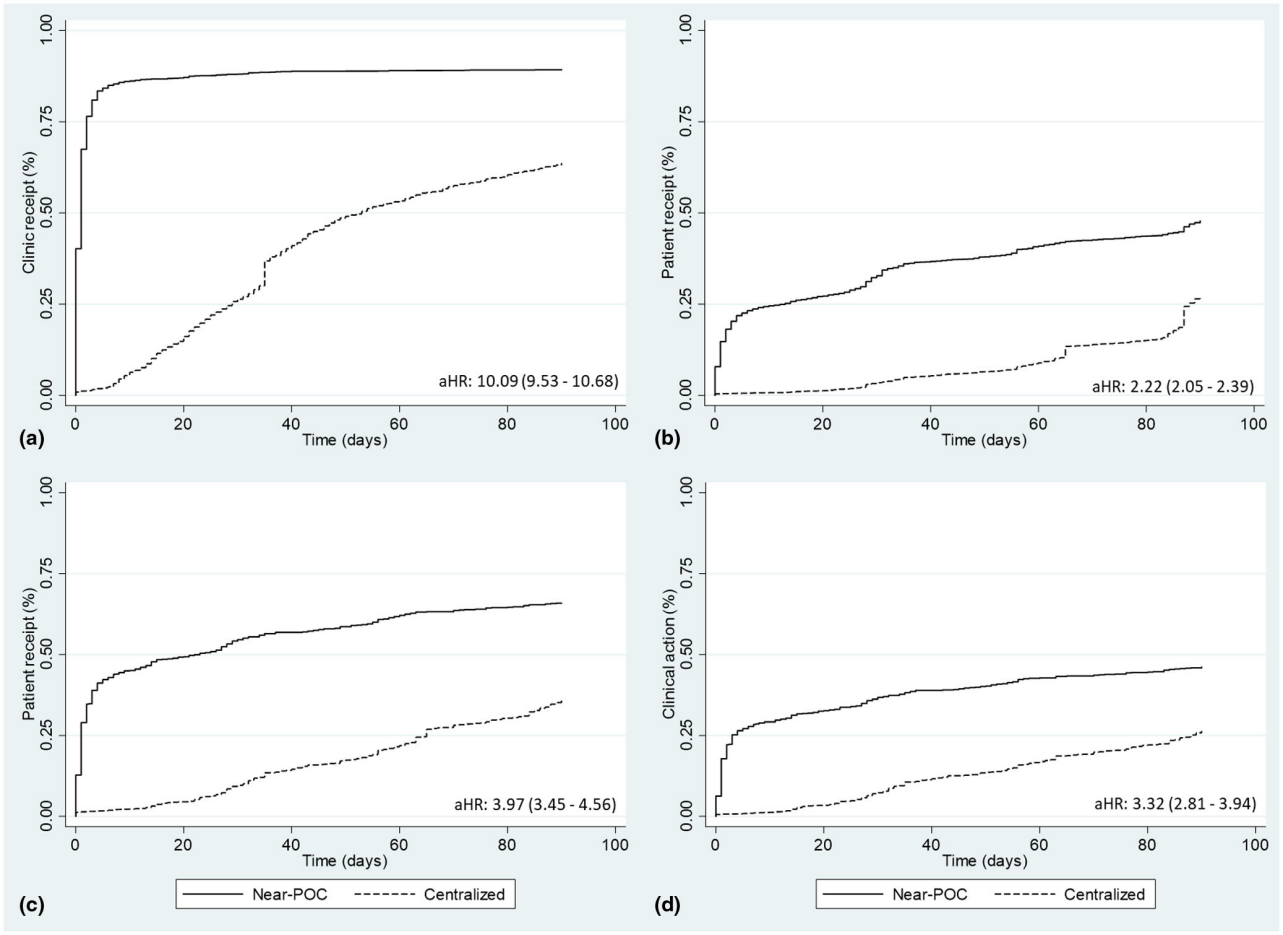
Kaplan‐Meier curves showing (**a**) receipt of results at the health clinic; (**b**) receipt of results by patients; (**c**) receipt of elevated HIV VL results by patients (**d**) clinical action of elevated HIV VL results, all for near‐POC HIV VL testing compared to centralized laboratory testing.

The median turnaround time from sample collection to clinic receipt of results was 1 day (IQR: 0 to 1) for near‐POC VL compared to 35 days (IQR: 20 to 48) for centralized VL testing (effect size: −38.7%, 95% CI: −47.2% to −30.3%, representing the likelihood that the turnaround time for near‐POC is less than centralized laboratory) (Table 3). Forty percent of near‐POC VL results were available at the clinic on the same day as testing. For near‐POC VL, there was almost no difference in the turnaround time from sample collection to clinic receipt of results for patients with elevated VL (0 days [IQR: 0 to 1]) versus suppressed VL (1 day [IQR 0 to 2]). Overall, 93% of near‐POC results were received by the clinic within 90 days of sample collection, compared to 64% when tested at central laboratory (aHR: 10.09, 95% CI: 9.53 to 10.68; Figure 1). For near‐POC testing, the highest rate of return to clinic was in DRC (100%) and the lowest was in Cameroon where there were data documentation issues (22%) (Table S2).

**Table 3:** Turnaround times and proportion of results received by the clinic and patient according to test location, either near‐POC or centralized lab

	Near‐POC		Centralized		Adjusted HR/effect size (95% CI)^a^
	N	n (%)/median (IQR)	N	n (%)/median (IQR)	
Sample collection to clinic receipt					10.09 (9.53 to 10.68)
Turnaround time (days)	3446	1 (0 to 1)	9734	35 (20 to 48)	−38.7% (−47.2% to −30.3%)
Proportion result received on same day	3879	1559 (40%)	15378	144 (1%)	
Proportion result received within seven days	3879	3310 (85%)	15378	505 (3%)	
Proportion result received within 30 days	3879	3416 (88%)	15378	4053 (26%)	
Proportion result received within 90 days	3594	3329 (93%)	15271	9734 (64%)	
Sample collection to patient receipt					2.22 (2.05 to 2.39)
Turnaround time (days)	1794	6 (1 to 33)	4172	68 (54 to 87)	−32.2% (−41.0% to −23.4%)
Proportion result received on same day	3879	305 (8%)	15378	63 (0.4%)	
Proportion result received within seven days	3879	919 (24%)	15378	105 (1%)	
Proportion result received within 30 days	3879	1271 (33%)	15378	537 (3%)	
Proportion result received within 90 days	3594	1781 (50%)	15271	4172 (27%)	
Sample collection to patient receipt, elevated patients					3.97 (3.45 to 4.56)
Turnaround time (days)	595	2 (1 to 15)	807	53 (29 to 65)	−37.7% (−45.9% to −29.4%)
Proportion result received on same day	925	118 (13%)	2276	29 (1%)	
Proportion result received within seven days	925	406 (44%)	2276	48 (2%)	
Proportion result received within 30 days	925	505 (55%)	2276	218 (10%)	
Proportion result received within 90 days	858	586 (68%)	2241	807 (36%)	
Sample collection to clinical action, elevated patients					3.32 (2.81 to 3.94)
Turnaround time (days)	418	3 (1 to 25)	593	49 (29 to 70)	−35.4% (−46.0% to −24.8%)
Proportion result received on same day	925	58 (6%)	2276	14 (1%)	
Proportion with clinical action within seven days	925	263 (28%)	2276	25 (1%)	
Proportion with clinical action within 30 days	925	340 (37%)	2276	167 (7%)	
Proportion with clinical action within 90 days	858	410 (48%)	2241	593 (26%)	
Sample collection to clinical action, children					2.88 (1.66 to 5.00)
Proportion with clinical action within 90 days	143	54 (38%)	230	46 (20%)	
Sample collection to clinical action, PBFW cohort only					11.56 (5.20 to 25.72)
Proportion with clinical action within 90 days	64	48 (75%)	64	9 (14%)	

ART, antiretroviral therapy; CI, confidence interval; HR, hazard ratio; IQR, interquartile range; PBFW, pregnant/breastfeeding women; POC, point‐of‐care.

^a^ For continuous outcomes, effect sizes were calculated using the Somers’ D test, accounting for facility‐level clustering. With this type of model, the effect size represents the likelihood that the turnaround time for near‐POC testing is greater than (if positive) or less than (if negative) the turnaround time for centralized laboratory testing. For time‐to‐event outcomes, maximum likelihood estimation was used for parametric regression survival‐time models with shared frailties to account for facility‐level clustering. Hazard ratios are adjusted for study design, sex, age, test reason, viral load suppression and country.

The median turnaround time from VL sample collection to patient receipt of results was six days (1 to 33) for near‐POC compared to 68 days (54 to 87) for centralized testing (effect size: −32.2%, 95% CI: −41.0% to −23.4% , Table 3). We did observe a high degree of heterogeneity by country. In Cameroon, Malawi and Senegal, the median turnaround time was one day, whereas in Kenya and Tanzania despite the turnaround time to the clinic of one to two days, the turnaround to the patient was 29 and 30 days respectively.

Across countries, within 30 days, 33% of patients had received results with near‐POC VL compared to only 3% with centralized. Within 90 days, 50% had received results when tested by near‐POC VL compared to 27% with centralized (aHR: 2.22, 95% CI: 2.05 to 2.39). Near‐POC results that were available at the clinic on the same day as sample collection were almost twice as likely to be returned to the patient within the 90 days of follow‐up (aHR: 1.88, 95% CI: 1.71 to 2.08).

Time to patient receipt also varied according to VL result. For near‐POC VL tests, patients with an elevated VL had a median time to result receipt of two days (IQR: 1 to 15) compared to 21 days (IQR: 1 to 47) for suppressed patients. Similar trends were observed for centralized laboratory testing with a median time of 53 days (IQR: 29 to 65) versus 83 days (IQR: 59 to 87) for patients with elevated VL compared to suppressed respectively.

**Figure 2:**
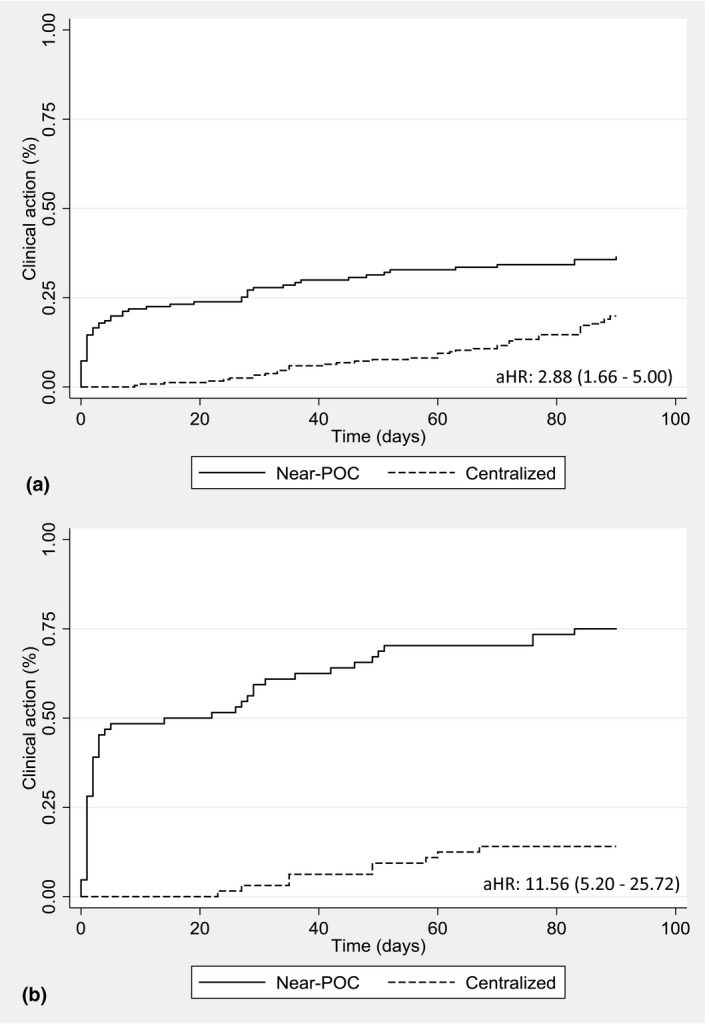
Kaplan‐Meier curve showing clinical action taken for (**a**) children and (**b**) pregnant and breastfeeding women with an elevated HIV VL monitored using near‐POC HIV VL testing compared to centralized laboratory testing.

The turnaround time from sample collection to clinical action for patients with elevated VL was three days (1 to 25) for near‐POC compared to 49 days (29 to 70) for centralized testing (effect size: −35.4%, 95% CI: −46.0% to −24.8%). Overall, only 37% of patients with elevated VL had received a clinical action within 30 days of sample collection for near‐POC compared to 7% for centralized testing (aHR: 3.32; 2.81 to 3.94). With near‐POC, when results were available at the clinic on the same day as sample collection, patients were 1.45 times more likely to have a clinical action (95% CI: 1.15 to 1.82). For both pregnant and breastfeeding women and children (under age 15 years) with elevated VL, use of near‐POC VL was associated with a significantly higher rate of clinical action within 90 days than centralized testing (aHR: 11.56; 95% CI: 5.20 to 25.72 and 2.88, 95% CI: 1.66 to 5.00 respectively) (Figure 2).

## 4 DISCUSSION

This multi‐country assessment shows that near‐POC VL implementation was consistently associated with significantly shorter turnaround times from sample collection to clinic receipt of results, patient receipt of results and clinical action in comparison to centralized testing. Among patients tested on near‐POC, if results were available on the same day, patients were twice as likely to receive their results and 1.45 times as likely to receive a clinical action as well. However, despite the demonstrated benefits of near‐POC VL, only 48% of patients with an elevated VL result received a clinical action during the 90 days of follow‐up, even though nearly half (40%) of near‐POC test results were available at the clinic on the same day. Therefore, the provision of near‐POC VL testing alone is not enough and more efforts are needed to ensure that timely results translate into improved clinical decision making for patients.

We did observe a significant impact of near‐POC VL on clinical action within high‐risk groups. Pregnant and breastfeeding women as well as children with elevated VL were significantly more likely to receive a clinical action when tested using near‐POC compared to centralized testing, suggesting that these high‐risk groups would benefit substantially from same day result delivery.

It is critical to ensure timely result delivery to all patients, including those with suppressed VL, as data has shown that knowing one’s VL improves retention [15]. While the data demonstrated results delivery to patients with elevated VL was expedited compared to patients with suppressed VL for both near‐POC and centralized laboratory tests, suppressed patients tested with near‐POC VL were not only more likely to receive their results than those tested with centralized VL but they also received results more quickly. Thus, where near‐POC VL testing can be offered for routine VL monitoring, it is likely to have a positive impact among suppressed patients as well. Moreover, this analysis excluded missing results, and given that there were more missing results for centralized VL testing, the true impact of near‐POC VL on results delivery may be even greater than estimated here.

Our findings of improved outcomes with near‐POC are consistent with the limited previously published research available. In a recent trial in South Africa, reduced turnaround times to result receipt by patient and appropriate switch to second‐line therapy with near‐POC was also observed [15]. However, the outcomes for both arms of that study were better than what was observed in our analysis (e.g. median time to patient receipt of results was 28 days for centralized VL and 0 days for POC VL, versus 68 days for centralized VL and 6 for near‐POC VL in our study), possibly due to South Africa’s stronger laboratory/clinical systems, the limited number of clinics included, and that the intervention included task‐shifting. In comparison, we observed larger gaps in outcomes in the POC arm in this multi‐country analysis, which may be a more accurate reflection of real‐world implementation across settings. In a descriptive assessment of a POC VL monitoring programme at decentralized Médecins Sans Frontières‐supported clinics in rural Malawi, 88% of test results were reviewed on the same day as sample collection; of patients with treatment failure, 86% switched to second line during follow‐up [11]. Another descriptive assessment of POC VL implementation in two clinics in Malawi reported that 78% of patients with a high VL result had a recorded clinical action, and 82% of patients received a clinical action on the same day as sample collection [12]. Other recently published studies and pilots in Africa concluded that POC VL testing was feasible [16, 17] and reduced turnaround times [18]. In summary, the published literature reports the benefits of POC VL testing across settings.

To close gaps in result utilization and improved patient outcomes, a number of systems gaps within programmes were identified for improvement, including result documentation at facilities, patient result communication and follow‐up and clinical acumen and capacity to make switches to second‐line therapy. In many of the health facilities across country settings, we noted poor documentation of patient receipt of results and more importantly, a lack of documentation of clinical actions taken for patients with elevated VL. Poor documentation of results makes it difficult for clinicians to efficiently identify which patients have not yet received their results or started adherence counselling or other follow‐up procedures. Further emphasis on the importance of maintaining accurate records of patient receipt of results and clinical actions taken will improve programme organization and the ability to flag patients who still need follow‐up. Feedback from programme staff indicated that many health facilities did not take proactive measures to return results to patients promptly. This was seen particularly in Kenya and Tanzania, where the time to patient receipt of results was substantially longer than in other settings, even for near‐POC VL. This translates to missed opportunities to provide timely clinical care for patients with an elevated VL, regardless of testing platform. Stronger patient communication and follow‐up are needed. This may take the form of automated SMS notifications or phone calls from the health facility when results are available. These approaches have been shown in numerous studies to be effective in retaining patients in care [19, 20, 21]. Additionally, stable patients with suppressed VL can be moved into a differentiated service delivery model of care to decongest facilities, allowing additional time to manage high‐risk populations.

This analysis has a number of limitations. First, this work was not designed as a robust study, nor was study design consistent, with the pre/post and cross‐sectional study designs utilized. Three of the studies had no comparison to centralized testing and therefore analyses were entirely descriptive in nature. The government in each country selected facilities to receive near‐POC devices, and this selection process may have introduced bias into study results. There is the potential that other factors changing over time at facilities influenced findings, and/or that differences in patients prioritized for near‐POC VL testing versus centralized testing influenced the outcomes observed, but sensitivity analyses limited to high‐risk groups found similar differences between near‐POC and centralized testing for all outcomes. Senegal only had 30 days of follow‐up, which most likely underestimates their results in comparison to the other countries. Every country also collected data for a different number of months, which could bias comparisons. The data quality in this assessment was limited by what was available in routinely collected register and patient record data available at health facilities. Some of the secondary analyses had relatively small sample sizes. The clinical action‐outcome could have been affected as staff received clinical training in preparation for near‐POC introduction, but not before in the centralized testing arms. We were not able to explore different clinical actions, transmission, qualitative patient impacts or long‐term follow‐up as outcomes. Finally, implementation practices varied across settings, although this can also be viewed as a strength of this analysis, which, along with the large number of patients tested overall across 57 public sector facilities in seven countries, suggests that findings may be generalizable to implementation in other settings in sub‐Saharan Africa.

## 5 CONCLUSIONS

In this large analysis across seven countries, near‐POC VL was associated with improvements in result availability at the clinic, as well as turnaround times to result availability and clinical action. High‐risk populations were prioritized for near‐POC VL and were more likely to receive clinical action. However, in many cases, test results did not get back to the patient and/or were not acted upon by clinicians. To truly capitalize on VL implementation and especially near‐POC VL, mentoring activities and support for strong facility data systems and patient follow‐up procedures are needed to improve outcomes for high‐risk patients as well as for routine patient management, ultimately reducing morbidity and mortality.

## COMPETING INTERESTS

The authors declare no competing interests.

## AUTHORS’ CONTRIBUTIONS

SK, LV, JAS, TP, ND and CEB designed the study and wrote the study protocol. MW, SK, CA, CB, BK, TM, JM, JN and MRR led study implementation. JJ and SK led the data analysis. CEB wrote the original draft of the manuscript. All authors critically reviewed the analysis and manuscript and approved the final version.

## Supporting information

Supplementary Material
