# Supplementary material for: Evaluation of near point‐of‐care viral load implementation in public health facilities across seven countries in sub‐Saharan Africa
Source: J Int AIDS Soc. 2021 Jan 16;24(1):e25663. doi: 10.1002/jia2.25663 (PMC7811577; doi:10.1002/jia2.25663)
Supplement: Supplementary file 1 — Supplementary Material [file JIA2-24-e25663-s001.docx]

**Supplemental Table 1. Characteristics of patients receiving HIV VL testing, by country and study design**

|  | **Near-POC** | | **Centralized** | |
| --- | --- | --- | --- | --- |
|  | **N** | **n (%) / median (IQR)** | **N** | **n (%) / median (IQR)** |
| **Valid Results** |  |  |  |  |
| Cameroon | 1525 | 1495 (98%) | 0 | 0 |
| DRC | 678 | 653 (96%) | 0 | 0 |
| Kenya | 316 | 194 (61%) | 463 | 462 (100%) |
| Malawi | 647 | 610 (94%) | 7128 | 5645 (79%) |
| Senegal-1 | 201 | 185 (92%) | 0 | 0 |
| Senegal-2 | 287 | 285 (99%) | 107 | 107 (100%) |
| Tanzania | 1089 | 1027 (94%) | 3927 | 3908 (100%) |
| Zimbabwe-1 | 1126 | 925 (82%) | 3351 | 2956 (88%) |
| Zimbabwe-2 | 926 | 838 (90%) | 2638 | 2300 (87%) |
| **Median Age** |  |  |  |  |
| Cameroon | 1525 | 38 (28 - 47) | - | - |
| DRC | 677 | 48 (41 - 55) | - | - |
| Kenya | 85 | 17 (11 - 30) | 462 | 16 (11 - 29) |
| Malawi | 484 | 38 (23 - 46) | 963 | 37 (27 - 45) |
| Senegal-1 | 189 | 40 (30 - 49) |  |  |
| Senegal-2 | 285 | 41 (33 - 50) | 104 | 38.5 (23 - 48.5) |
| Tanzania | 1052 | 40 (21 - 49) | 3910 | 45 (37 - 53) |
| Zimbabwe-1 | 1058 | 36 (27 - 44) | 3029 | 40 (33 - 48) |
| Zimbabwe-2 | 870 | 37 (27 - 45) | 2593 | 43 (36 - 50) |
| **Sex, female** |  |  |  |  |
| Cameroon | 1509 | 1101 (72%) | - | - |
| DRC | 678 | 513 (76%) | - | - |
| Kenya | 312 | 291 (342%) | 460 | 331 (72%) |
| Malawi | 639 | 354 (73%) | 7007 | 4500 (64%) |
| Senegal-1 | 201 | 159 (84%) |  |  |
| Senegal-2 | 287 | 216 (76%) | 107 | 78 (73%) |
| Tanzania | 1085 | 728 (69%) | 3924 | 2775 (71%) |
| Zimbabwe-1 | 1122 | 831 (79%) | 3280 | 2047 (62%) |
| Zimbabwe-2 | 926 | 608 (70%) | 2629 | 1595 (61%) |
| **Elevated VL (≥1000 copies/ml)** |  |  |  |  |
| Cameroon | 1495 | 348 (23%) | 0 | 0 |
| DRC | 653 | 155 (24%) | 0 | 0 |
| Kenya | 194 | 36 (19%) | 462 | 121 (26%) |
| Malawi | 610 | 303 (50%) | 5645 | 750 (13%) |
| Senegal-1 | 185 | 59 (32%) | 0 | 0 |
| Senegal-2 | 285 | 67 (24%) | 107 | 35 (33%) |
| Tanzania | 1027 | 84 (8%) | 3908 | 553 (14%) |
| Zimbabwe-1 | 925 | 293 (32%) | 2956 | 611 (21%) |
| Zimbabwe-2 | 838 | 142 (17%) | 2300 | 206 (9%) |
| VL: viral load; POC: point-of-care; IQR: interquartile range; DRC: Democratic Republic of Congo | | | | |

**Supplemental Table 2. Point-of-care HIV VL in relation to caregiver receipt of results and follow-up clinical action, by country and study design**

|  | **Near-POC** | | **Centralized** | |  |
| --- | --- | --- | --- | --- | --- |
|  | **n** | **% (95% CI) / median (IQR)** | **n** | **% (95% CI) / median (IQR)** | **Effect size (95% CI)**^†^ |
| **Turnaround time (days), Sample Collection to Patient Receipt** | | | | | |
| Cameroon | 73 | 1 (0 - 4) |  |  |  |
| DRC | 652 | 2 (1 - 3) |  |  |  |
| Kenya | 99 | 29 (26 - 56) | 202 | 56 (38 - 63) | **18.2% (8.6% - 27.8%)** |
| Malawi | 220 | 1 (0 - 5) | 1303 | 57 (33 - 81) | **22.9% (13.7% - 32.1%)** |
| Senegal-1 | 61 | 1 (1 - 1) |  |  |  |
| Senegal-2 | 13 | 3 (2 - 12) | 0 | na (na - na) |  |
| Tanzania | 449 | 30 (19 - 46) | 575 | 65 (65 - 65) | **56.4% (30.2% - 82.7%)** |
| Zimbabwe-1 | 534 | 2 (1 - 8) | 1278 | 87 (86 - 87) | **50.8% (26.9% - 74.6%)** |
| Zimbabwe-2 | 479 | 5 (1 - 47) | 814 | 84 (59 - 87) | **36.2% (24.3% - 48.1%)** |
| **Turnaround time (days), Sample Collection to Clinical Action, Elevated Patients** | | | | | |
| Cameroon | 0 | na (na - na) |  |  |  |
| DRC | 1 | 6 (6 - 6) |  |  |  |
| Kenya | 7 | 29 (27 - 49) | 26 | 54.5 (35 - 72) | **12.6% (1.8% - 23.5%)** |
| Malawi | 67 | 2 (0 - 18) | 187 | 56 (33 - 70) | **30.2% (20.8% - 39.7%)** |
| Senegal-1 | 14 | 1 (1 - 2) |  |  |  |
| Senegal-2 | 8 | 3 (2 - 12.5) | 0 | na (na - na) |  |
| Tanzania | 22 | 45 (28 - 56) | 86 | 63 (49 - 84) | **26.8% (9.6% - 43.9%)** |
| Zimbabwe-1 | 236 | 2 (1 - 5) | 249 | 32 (23 - 53) | **47.7% (37.1% - 58.3%)** |
| Zimbabwe-2 | 78 | 28 (2 - 52) | 45 | 71 (40 - 87) | **30.3% (15.3% - 45.4%)** |

^†^For continuous outcomes, effect sizes were calculated using the Somers’ D test, accounting for facility-level clustering. With this type of model, the effect size represents the likelihood that the turnaround time for near-POC testing is greater than (if positive) or less than (if negative) the turnaround time for centralized laboratory testing.

VL: viral load; POC: point-of-care; IQR: interquartile range; CI: confidence interval; DRC: Democratic Republic of Congo
